# Supplementary material for: Microstructural Properties of the Cerebellar Peduncles in Children With Developmental Language Disorder
Source: Neurobiol Lang (Camb). 2024 Aug 15;5(3):774–94. doi: 10.1162/nol_a_00142 (PMC11338306; doi:10.1162/nol_a_00142)

Supplementary Table (S1): List of tests used as part of the neuropsychological battery. The measures under the *Language* and *Memory* domains were summarized into two factors using factor analysis.

Domain	Skill	Test
Language	Receptive Grammar	Test for Reception of Grammar TROG-2; Bishop, 2003
	Expressive Grammar	Clinical Evaluation of Language Fundamentals CELF-4 Sentence recall; Semel et al., 2004
	Receptive Vocabulary	Receptive One-Word Picture Vocabulary Test ROWPVT-4; Martin and Brownell, 2011
	Expressive Vocabulary	Expressive One-Word Picture Vocabulary Test EOWPVT-4; Martin and Brownell, 2011
	Narrative Production & Comprehension	Expression, Reception and Recall of Narrative Instrument ERNNI; Bishop, 2004
	Phonological Processing	Nonword repetition; Snowling et al., 2015
Reading	Decoding & Word Reading	Test Of Word Reading Efficiency TOWRE; Torgesen et al., 1999
Memory	Short-term & Working Memory	Forward and Backward Digit Span Children's Memory Scale CMS; Cohen, 1997
	Episodic Auditory-Verbal Learning	Word lists CMS; Cohen, 1997
Motor	Oromotor Coordination	Oromotor sequences subtest of the NEPSY (A Developmental NEuroPSYchological assessment); Korkman et al. 1998
	Gross and fine Motor Dexterity	Purdue Pegboard; Tiffin, 1968
Non-verbal IQ	Visuospatial Ability	Block Design Wechsler Intelligence Scale for Children WISC IV; Wechsler, 2004
	Visual/abstract/perceptual Reasoning	Matrix Reasoning WISC IV; Wechsler, 2004
	Processing speed	Coding WISC IV; Wechsler, 2004

Supplementary Table (S2): Mean tract FA of cerebellar peduncles (CP) in each group

	**TD** (N=77)	**DLD** (N=54)	**HSL** (N=28)
**Left Inferior CP**			
Mean (SD)	0.45 (0.06)	0.42 (0.06)	0.44 (0.06)
Range (Min - Max)	0.30 - 0.56	0.27 - 0.53	0.31 - 0.56
**Right Inferior CP**			
Mean (SD)	0.47 (0.04)	0.45 (0.05)	0.46 (0.05)
Range (Min - Max)	0.33 - 0.57	0.31 - 0.55	0.33 - 0.53
**Left Superior CP**			
Mean (SD)	0.43 (0.03)	0.41 (0.03)	0.44 (0.03)
Range (Min - Max)	0.38 - 0.51	0.34 - 0.48	0.39 - 0.54
**Right Superior CP**			
Mean (SD)	0.42 (0.04)	0.41 (0.03)	0.43 (0.03)
Range (Min - Max)	0.34 - 0.51	0.34 - 0.49	0.37 - 0.48
**Middle CP**			
Mean (SD)	0.43 (0.04)	0.42 (0.04)	0.42 (0.03)
Range (Min - Max)	0.29 - 0.50	0.33 - 0.52	0.34 - 0.49

Supplementary Table (S3): Model summaries for fractional anisotropy (FA) in the inferior cerebellar peduncles (ICP) when excluding HSL group from the analysis.

	ICP Model			ICP Model including Age, Sex, Motion		
Predictor	Beta	95% CI[1]	p-value	Beta	95% CI[1]	p-value
group						
TD	—	—		—	—	
DLD	-0.09	-0.17, -0.02	0.013	-0.08	-0.16, -0.01	0.025
hemisphere						
l	—	—		—	—	
r	0.09	0.03, 0.15	0.002	0.09	0.03, 0.15	0.002
whole_brain_FA	1.6	-1.8, 5.0	0.4	1.7	-1.8, 5.1	0.3
group * hemisphere						
DLD * r	0.03	-0.05, 0.12	0.4	0.03	-0.05, 0.12	0.4
ageInYears				0.00	-0.01, 0.02	0.6
sex						
Male				—	—	
Female				0.08	0.02, 0.14	0.006
relMotion				0.11	-0.13, 0.35	0.4

[1]CI = Confidence Interval

Supplementary Table (S4): Mean tract AD and RD in the inferior cerebellar peduncles (ICP) in each group.

	**TD** (N=77)	**DLD** (N=54)	**HSL** (N=28)
**AD**			
**Left ICP**			
Mean (SD)	0.0011855 (0.0000600)	0.0011571 (0.0000552)	0.0011627 (0.0000524)
Range (Min - Max)	0.0010160 - 0.0013100	0.0010270 - 0.0012950	0.0010560 - 0.0012560
**Right ICP**			
Mean (SD)	0.0011915 (0.0000478)	0.0011771 (0.0000531)	0.0011904 (0.0000518)
Range (Min - Max)	0.0010440 - 0.0013530	0.0010560 - 0.0013340	0.0010570 - 0.0013060
**RD**			
**Left ICP**			
Mean (SD)	0.0005752 (0.0000603)	0.0005894 (0.0000614)	0.0005794 (0.0000693)
Range (Min - Max)	0.0004640 - 0.0007280	0.0004870 - 0.0007100	0.0004570 - 0.0007760
**Right ICP**			
Mean (SD)	0.0005485 (0.0000403)	0.0005628 (0.0000524)	0.0005570 (0.0000507)
Range (Min - Max)	0.0004570 - 0.0006790	0.0004680 - 0.0007560	0.0004730 - 0.0007200

Supplementary Figure (S5): Mean Axial Diffusivity (AD) in the inferior cerebellar peduncles (ICP) by group (TD = typically developing in blue, DLD = developmental language disorder in yellow, HSL = history of speech and language impairments in green) and across hemispheres. *Note:* The box is drawn from first to third quartile with the horizontal line drawn in the middle to denote median FA. Whiskers show 1.5*IQR with data beyond the end of the whiskers (group outliers) being plotted individually.

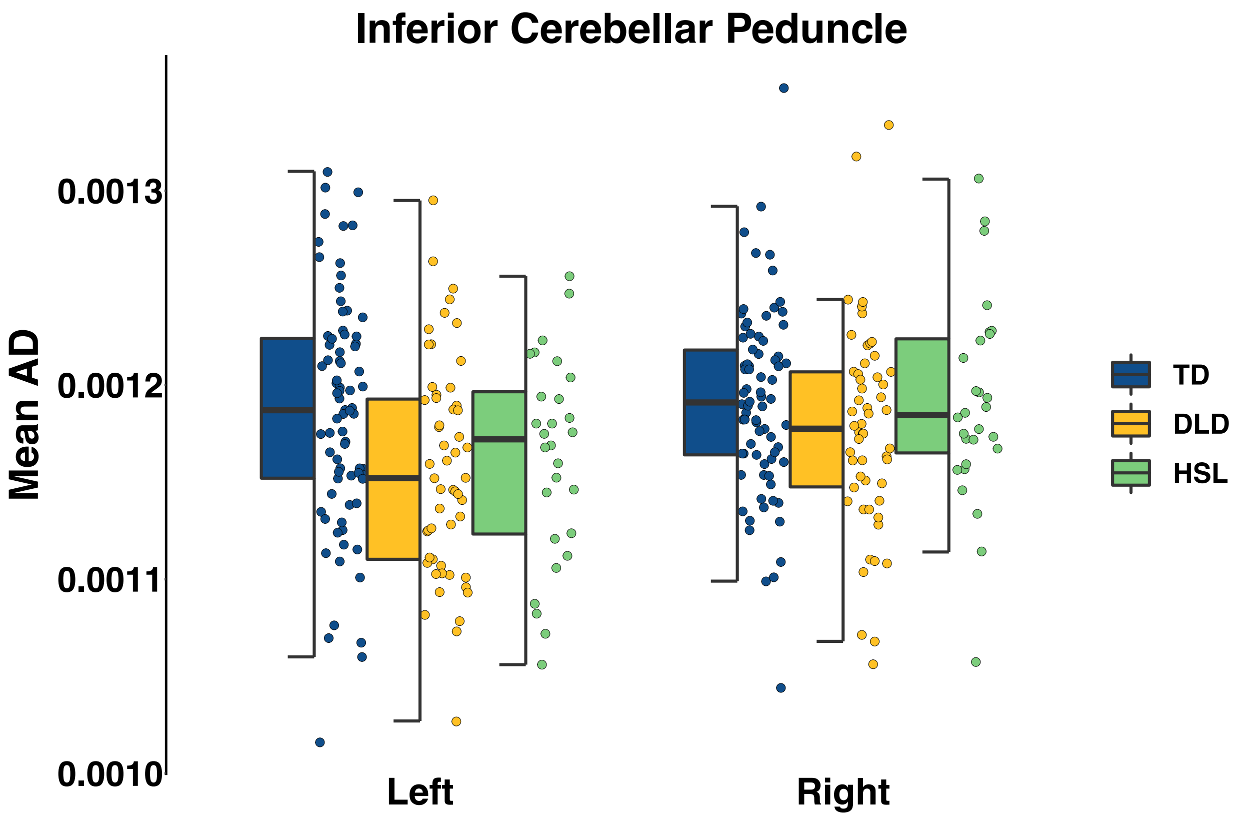

Supplementary Table (S6): Mean tract NODDI measures [orientation dispersion (OD), fraction of the data that is isotropic (f_{iso}), and fraction of intra-cellular compartment compared to the total intra- and extra-cellular compartment (f_{intra})] in the inferior cerebellar peduncles (ICP) in each group.

	**TD** (N=77)	**DLD** (N=54)	**HSL** (N=28)
**OD**			
**Left ICP**			
Mean (SD)	0.24 (0.04)	0.27 (0.04)	0.26 (0.05)
Range (Min - Max)	0.18 - 0.36	0.20 - 0.41	0.19 - 0.35
**Right ICP**			
Mean (SD)	0.23 (0.03)	0.24 (0.04)	0.23 (0.03)
Range (Min - Max)	0.18 - 0.34	0.19 - 0.36	0.19 - 0.32
**f_{intra}**			
**Left ICP**			
Mean (SD)	0.63 (0.03)	0.63 (0.03)	0.64 (0.03)
Range (Min - Max)	0.58 - 0.70	0.59 - 0.69	0.56 - 0.70
**Right ICP**			
Mean (SD)	0.63 (0.03)	0.63 (0.03)	0.63 (0.03)
Range (Min - Max)	0.57 - 0.68	0.56 - 0.70	0.59 - 0.70
**f_{iso}**			
**Left ICP**			
Mean (SD)	0.12 (0.02)	0.11 (0.02)	0.11 (0.02)
Range (Min - Max)	0.07 - 0.19	0.07 - 0.17	0.08 - 0.16
**Right ICP**			
Mean (SD)	0.11 (0.02)	0.11 (0.02)	0.11 (0.02)
Range (Min - Max)	0.08 - 0.18	0.07 - 0.18	0.07 - 0.15

Supplementary Figure (S7): Mean Orientation Dispersion (OD) in the inferior cerebellar peduncles (ICP) by group (TD = typically developing in blue, DLD = developmental language disorder in yellow, HSL = history of speech and language impairments in green) and across hemispheres. *Note:* The box is drawn from first to third quartile with the horizontal line drawn in the middle to denote median FA. Whiskers show 1.5*IQR with data beyond the end of the whiskers (group outliers) being plotted individually.

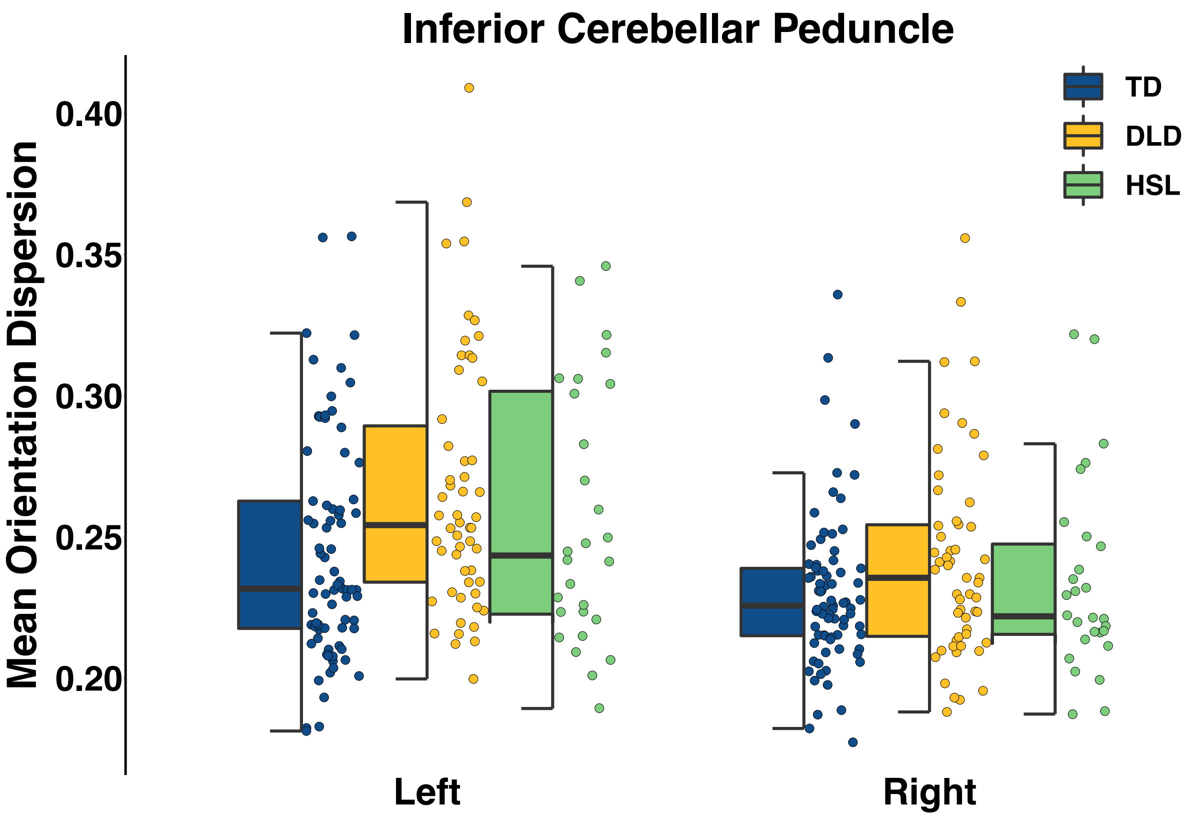

Supplementary Table (S8): Mean cerebellar volume (in mm^3) in DLD, HSL, and TD groups per tissue type (cortex, white matter) in each hemisphere.

	**DLD** (N=53)	**HSL** (N=27)	**TD** (N=74)
**Left Cerebellum Cortex**			
Mean (SD)	56326.60 (6184.66)	58572.56 (6763.65)	58072.65 (5503.35)
**Left Cerebellum White Matter**			
Mean (SD)	13683.67 (1836.93)	15418.46 (2730.48)	14838.51 (1643.63)
**Right Cerebellum Cortex**			
Mean (SD)	56864.42 (6503.32)	59617.42 (7560.55)	58767.31 (5710.00)
**Right Cerebellum White Matter**			
Mean (SD)	13051.03 (1663.48)	14727.45 (2110.11)	13997.30 (1528.11)
**ICV**			
Mean (SD)	1322159.64 (165825.79)	1416074.17 (184155.81)	1339592.88 (136502.63)

Supplementary Figure (S9): The thresholded inferior (ICP; left in red, right in blue), middle (MCP; green) and Superior (SCP; left in brown, right in yellow) cerebellar peduncles overlaid on the FSL_HCP065_FA image in a typical DLD (left) and HSL child (right).

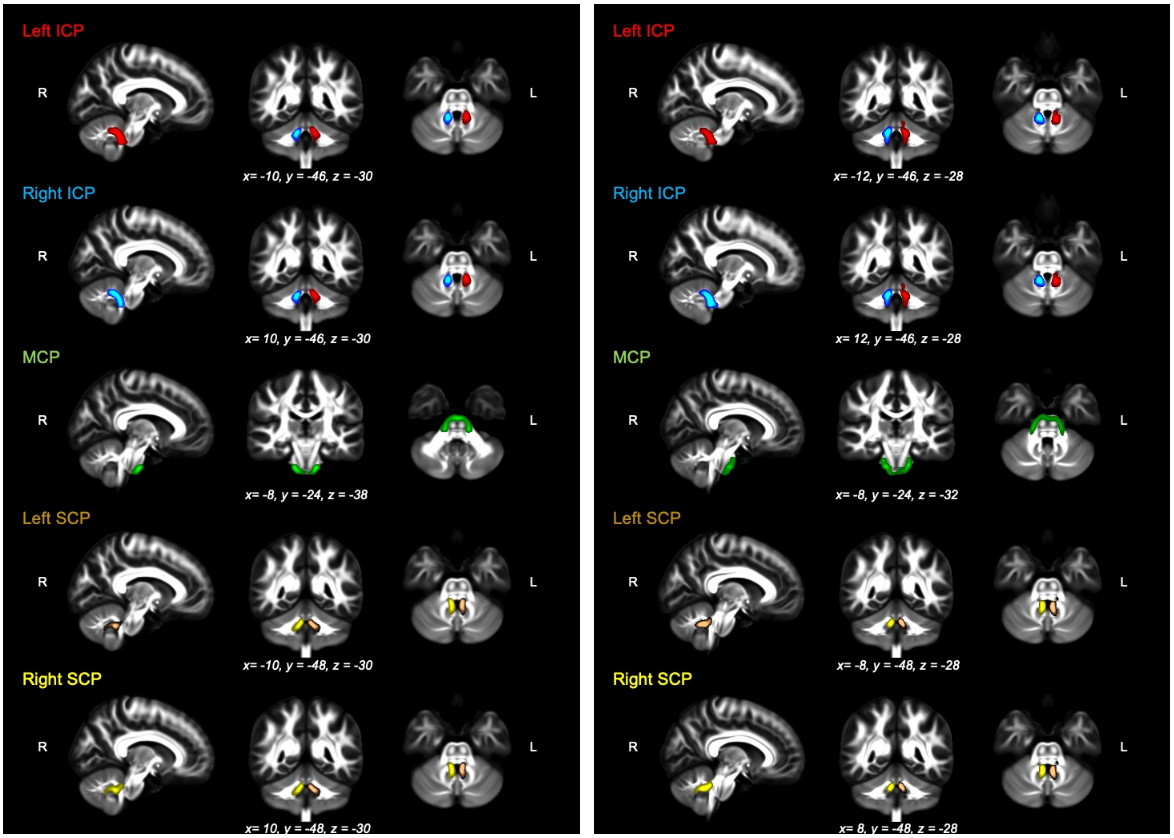

Supplement: Supplementary file 1 [file nol-5-3-774-s001.pdf]
